# Low-Voltage Zones as the Atrial Fibrillation Substrates: Relationship With Initiation, Perpetuation, and Termination

**DOI:** 10.3389/fcvm.2021.705510

**Published:** 2021-08-02

**Authors:** Zheng Liu, Yu Xia, Changyan Guo, Xiaofeng Li, Pihua Fang, Xiandong Yin, Xinchun Yang

**Affiliations:** ^1^Heart Center, Beijing Chaoyang Hospital, Capital Medical University, Beijing, China; ^2^State Key Laboratory of Cardiovascular Disease, Cardiac Arrhythmia Center, Fuwai Hospital, National Center for Cardiovascular Diseases, Chinese Academy of Medical Sciences and Peking Union Medical College, Beijing, China; ^3^Department of Cardiology, Xilin Gol League Central Hospital, Inner Mongolia, Xilinhot, China

**Keywords:** atrial fibrillation, electroanatomic mapping, low voltage, inducibility, sustainability, termination

## Abstract

**Background:** Low-voltage zones (LVZs) were usually targeted for ablation in atrial fibrillation (AF). However, its relationship with AF initiation, perpetuation, and termination remains to be studied. This study aimed to explore such relationships.

**Methods:** A total of 126 consecutive AF patients were enrolled, including 71 patients for AF induction protocol and 55 patients for AF termination protocol. Inducible and sustainable AF were defined as induced AF lasting over 30 and 300 s, respectively. Terminable AF was defined as those that could be terminated into sinus rhythm within 1 h after ibutilide administration. Voltage mapping was performed in sinus rhythm for all patients. LVZ was quantified as the percentage of the LVZ area (LVZ%) to the left atrium surface area.

**Results:** The rates of inducible, sustainable, and terminable AF were 29.6, 18.3, and 38.2%, respectively. Inducible AF patients had no significant difference in overall LVZ% compared with uninducible AF patients (10.2 ± 11.8 vs. 8.5 ± 12.6, *p* = 0.606), while sustainable and interminable AF patients had larger overall LVZ% than unsustainable (16.2 ± 11.5 vs. 0.5 ± 0.7, *p* < 0.001) and terminable AF patients (44.6 ± 26.4 vs. 26.3 ± 22.3, *p* < 0.05), respectively. The segmental LVZ distribution pattern was diverse in the different stages of AF. Segmental LVZ% difference was initially observed in the anterior wall for patients with inducible AF, and the septum was further affected in those with sustainable AF, and the roof, posterior wall, and floor were finally affected in those with interminable AF.

**Conclusions:** The associations between LVZ with AF initiation, perpetuation, and termination were different depending on its size and distribution.

## Introduction

Atrial fibrillation (AF) is the most common cardiac arrhythmia, with an increasing prevalence rate in recent years (1). Currently, it is believed that the presence of triggers and the remolding of atrial substrate are essential mechanisms for the occurrence of AF (1). The extension of atrial fibrosis reflecting the severity of atrial remolding affects long-term outcome in AF patients that underwent catheter ablation (2–4). Low-voltage zones (LVZ) harboring complex electrogram or colocalizing spatio-temporal dispersion are considered as surrogates for atrial fibrosis. Ablation targeting LVZ can help to terminate AF and improve long-term outcome for patients that underwent AF ablation (5–8). However, the specific relationships between the quantity and distribution of LVZ and the initiation, perpetuation, and termination of AF have not been studied systematically. In this study, we aimed to explore these relationships.

## Methods

### Study Population

**Figure 1:**
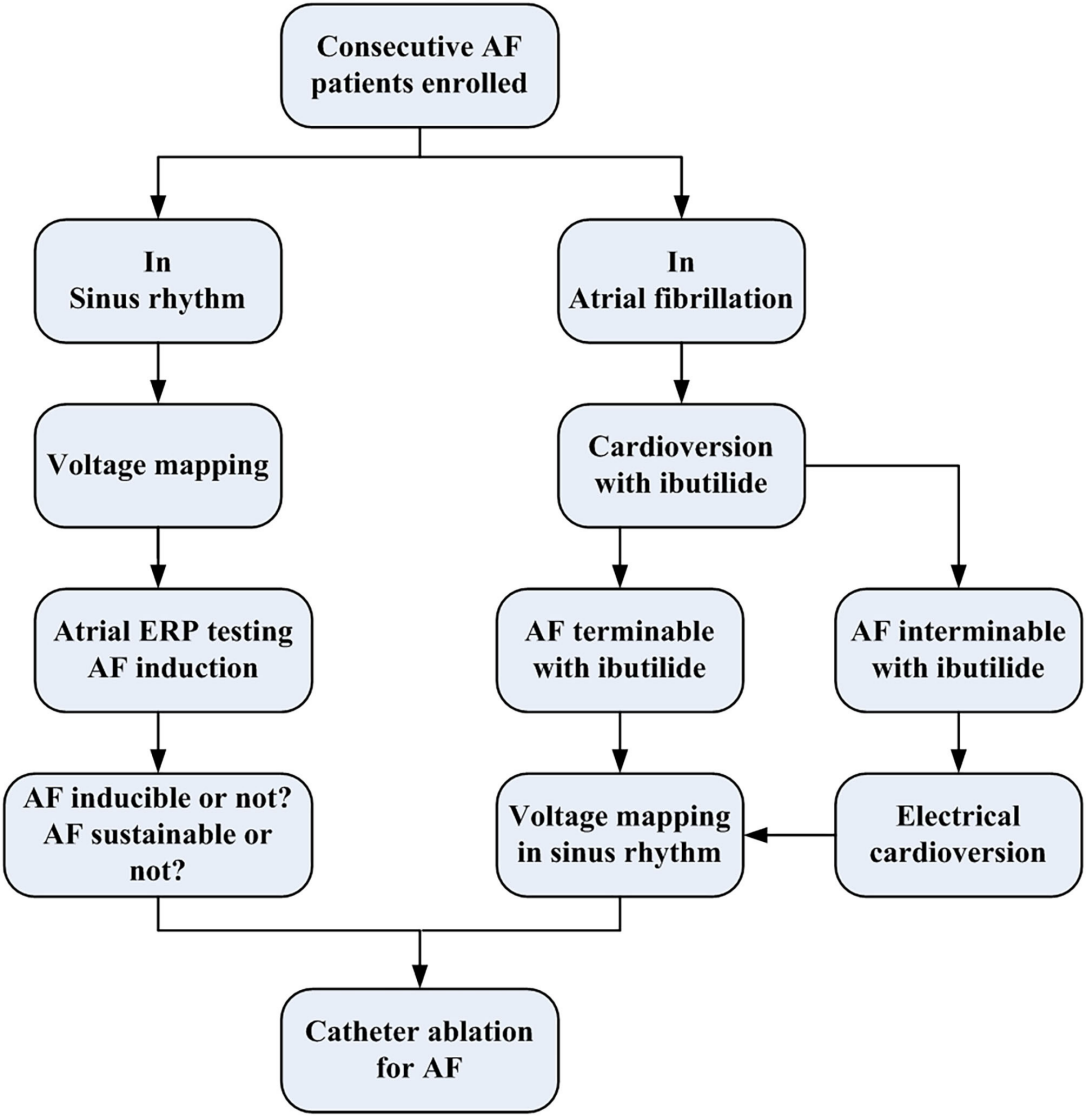
The flowchart of the present study protocol. AF, atrial fibrillation; ERP, effective refractory period.

Between November 2017 and November 2018, consecutive patients who underwent pulmonary vein isolation (PVI) procedure for non-valvular AF in our hospital were enrolled for analysis. After excluding (1) patients with amiodarone-taking history within 6 months that might affect the AF inducibility, (2) those who had previous catheter ablation or cardiac surgical procedure that might cause a scar in the left atrium (LA), (3) those with advanced heart failure or prolonged QTc (>450 ms), and (4) those who had contraindications to catheter ablation and anticoagulation. Finally, a total of 126 patients were analyzed, including 71 patients with sinus rhythm assessed for AF inducibility and sustainability by introducing atrial pacing and 55 patients with spontaneous AF assessed for termination with ibutilide administration. All patients underwent voltage mapping of the LA during sinus rhythm and AF dynamic assessment before PVI (Figure 1). Demographic factors, AF type, clinical mobilities [calculated as CHA_2_DS_2_-VASc score (1)], and cardiac ultrasonographic parameters were collected. The study protocol conforms to the ethical guidelines of the 1975 Declaration of Helsinki as reflected in a prior approval by the human research committee of the institution. All patients provided written informed consents before the clinical procedure.

### Electrophysiological Study

All antiarrhythmic drugs were ceased at least five half-lives before the procedure. The procedure was performed with sedation utilizing fentanyl. LA access was achieved after a transseptal puncture, with two long sheaths introduced into the LA. Intravenous heparin was given with 100 U/kg, adjusted with activated clotting time at 250–350 s before LA transseptal puncture. A 10-pole catheter with an interelectrode space of 2-5-2 mm was positioned in the coronary sinus with the proximal electrode pair near the ostium of the CS. A steerable five-spine 20-pole mapping catheter (PentaRay, Biosense Webster, USA; interelectrode spacing 2-6-2 mm) was advanced into the LA through the long sheath for mapping. Finally, a 3.5-mm tip, open irrigated, contact force-sensing catheter (ThermoCool SmartTouch, Biosense Webster, USA) was placed in the LA via the long sheath for mapping and ablation.

### Atrial Pacing Protocol

In patients with sinus rhythm, atrial effective refractory period (ERP) was evaluated at fixed pacing output of 10-mA and 2-ms pulse width, delivered in three sites including the anterior wall of the left and right pulmonary vein (PV) ostium and the ostium of the coronary sinus vein by using the contact force sensing catheter. A 5–10-g electrode–tissue contact force was required to initiate pacing in order to ensure enough but not excessive contact. Pacing protocol was the same as that described by Sanders et al. (9), which contained an extra stimulus introduced following an eight-beat drive with a cycle length of 500 ms, starting with a start extra stimulus coupling interval of 150 ms and increasing in 10-ms increments. Local ERP was defined as the shortest coupling interval that was able to capture the LA at the pacing site. At each site, the ERP was measured three times, and the average value was used for analysis. ERP mean was defined as the mean value of the local ERP of three pacing sites. ERP dispersion was defined as the difference between the longest and the shortest local ERP of the three sites (10, 11). The inducibility of AF by single extra stimulus was counted during ERP determination. AF was defined as any rapid atrial activity (rate >350 beats/min) with irregular cycle length, polarity, configuration, and amplitude on atrial electrograms lasting over 5 cycles (11). The duration of induced AF was documented. Among multiple induced AF episodes in the same patients, the longest duration was analyzed. Inducible AF was defined only when AF lasts over 30 s. Sustainable AF was defined as those that last over 300 s as previously described (9). Incidence of induced AF of any duration, ≥30 s, and ≥300 s was counted. If induced AF could not terminate after 300 s simultaneously, electrical cardioversion was performed to restore sinus rhythm. After awaiting a period of 10 min, ERP measurement was continued.

### Spontaneous Atrial Fibrillation Termination Protocol

The ability to terminate spontaneous AF was assessed with 1 mg of ibutilide administered intravenously in 10 min with continuous electrocardiograph (ECG) monitoring during the whole procedure. If paired premature ventricular contrast or premature ventricular contrast with R-on-T phenomenon was observed, ibutilide administration was stopped immediately. If Torsade de points occurred, ibutilide administration was stopped immediately, administration with 1.25 g of magnesium sulfate and 100 mg of lidocaine was performed to prevent it from recurring. If hemodynamics were unstable or Torsade de points sustained over 30 s, electrical cardioversion was performed immediately to restore sinus rhythm. If AF could not be stopped after 1 h from the beginning of ibutilide administration, electrical cardioversion was performed to restore sinus rhythm to perform voltage mapping. Cardioversion from AF to sinus rhythm in 1 h was documented. If AF could be terminated within 1 h, terminable AF was defined; otherwise, interminable AF was defined. Due to ibutilide affection, we did not test ERP in patients with ibutilide termination protocol.

### Voltage Electroanatomic Mapping

**Figure 2:**
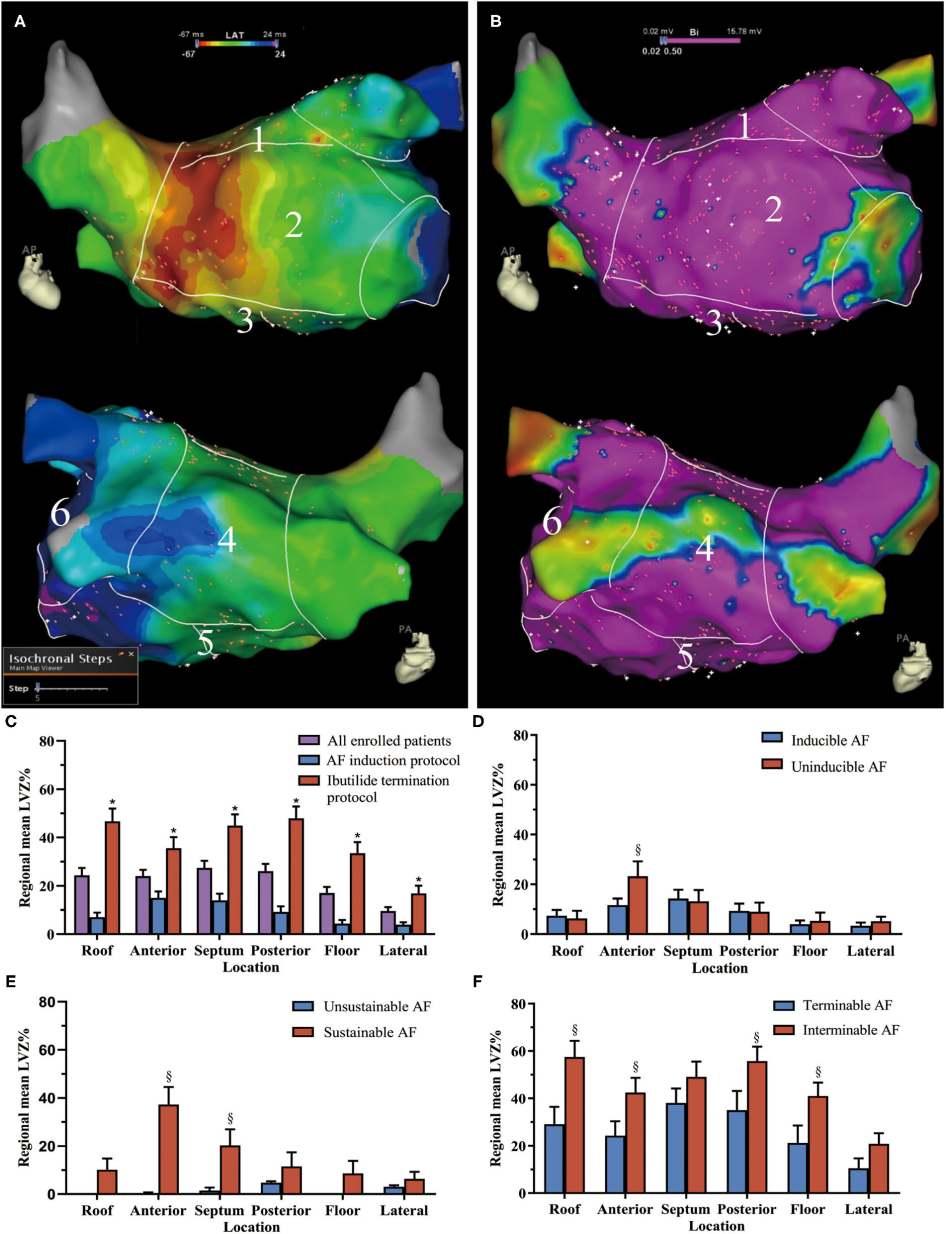
The low-voltage zone (LVZ) distribution in different atrial segments. Example for the six segments of **(A)** the left atrium (LA) in the activation map and **(B)** bipolar voltage map (1 = roof, 2 = anterior wall, 3 = septum, 4 = posterior wall, 5 = floor, and 6 = lateral wall). In the voltage map, areas in purple indicate healthy myocardia with sinus voltage ≥0.5 mV, areas with color range from red to blue indicate LVZ with voltage <0.5 mV. **(C)** The LVZ distribution in all enrolled patients and patients with different protocol. The comparison of LVZ distribution in the LA between **(D)** inducible and uninducible AF patients, **(E)** sustained and unsustained AF patients, and **(F)** interminable and terminable AF patients. *Compared between AF induction and ibutilide termination protocol, *p* < 0.05. ^§^Compared between patients with and without inducible, sustained, and terminable AF, *p* < 0.05. The bar charts were drawn using means and standard error. AF, atrial fibrillation; LVZ, low voltage zones; LA, left atrium.

An electroanatomic map of the LA and PVs was constructed. All LA voltages were collected in sinus rhythm before ablation. Mapping was performed with the PentaRay catheter and the contact force-sensing ablation catheter. To ensure detailed and uniform mapping of the entire chamber, we set the mapping filling threshold of 3 mm and require a minimum of 500 points for a complete map. When using PentaRay, “Tissue proximity indication” was switched on. The proximity to the tissue is indicated by highlighting the electrodes with a white frame. To ensure contact, the catheter was manipulated in consistent movement with the cardiac silhouettes under fluoroscopy. In the LA septum, where reliable contact cannot be achieved with the PentaRay catheter, the contact force-sensing ablation catheter was used, and a minimum contact force of 5 g was required. Annotation of the mitral annulus was tagged in the LA electroanatomic map where the A/V amplitude ratio is 1:1 as Jais et al. previously described (12). To avoid perforation of the LA appendage (LAA), catheters were prohibited from going deep into the LAA. Thus, surface area and volume of the LAA were not counted for analysis. Measurements of the LA surface area were performed by the tool inside the system with exclusion of the mitral annulus, LAA, and PVs. Volume of the LA was manually assessed with a tool included in the mapping system after exclusion of PV branches extending 1 cm over the PV ostium and LAA. All bipolar electrograms were filtered between 30 and 500 Hz. Local voltage was defined as the amplitude of the peak positive to the peak negative deflections. Low voltage was set to a cutoff <0.5 mV in sinus rhythm as commonly used. LVZ was defined if it possessed at least three neighboring sampled points with voltage <0.5 mV as previously described (13). LVZ area was measured with the same tool for LA surface measurement. The size of LVZ was presented as a percentage of the entire LA surface (LVZ%). To describe the regional distribution of the LVZ, LA was manually separated into six segments as previously described (Figures 2A,B) (8). Segmental LVZ% was measured as the ratio of the regional LVZ area to the segmental area.

### Statistical Analysis

Continuous variables were expressed as mean ± standard deviation. Categorical variables were expressed as counts (percentages). Independent samples *t*-test and Mann–Whitney *U*-test were used for the continuous variables according to their distributions. Chi-square test and Fisher's exact test were used for categorical variables. Univariate and multivariate logistic regression analyses were used to determine the factors that were associated with the inducibility, sustainability, and termination of AF before ablation. A *p*-value < 0.05 was considered statistically significant. The SPSS statistical package (version 22.0 for Windows; SPSS Inc, Chicago, IL, USA) was used for analyses.

## Results

### Characteristics of the Patients

A total of 126 AF patients (mean age: 66.3 ± 10.4 years, female: 39.6%, and persistent AF: 38.9%) were enrolled. Characteristics of the patients are shown in Table 1. The duration of diagnosed AF before procedure was 5 months [1, 25].

**Table 1:** Baseline characteristics of patients in induction and termination protocol.

	**All (*N* = 126)**	**Induction protocol(*N* = 71)**	**Termination protocol (*N* = 55)**	***p*-value**
Age (years)	66.3 ± 10.4	67.5 ± 9.3	64.9 ± 11.6	0.181
Female (%)	50 (39.6%)	34 (47.9%)	16 (21.9%)	0.032
Persistent AF (%)	49 (38.9%)	7 (9.9%)	42 (76.4%)	<0.001
CHA_2_DS_2_-VASc	3.3 ± 1.7	3.6 ± 1.7	3.0 ± 1.7	0.033
LA volume (ml)	146.2 ± 37.7	140.1 ± 41.3	154.3 ± 31.1	0.030
LA diameter (mm)	41.8 ± 5.5	39.8 ± 5.2	44.6 ± 4.9	<0.001
LVEF (%)	64.6 ± 7.7	64.9 ± 7.6	63.0 ± 8.4	0.187
ERP mean (ms)	214.1 ± 24.8	214.1 ± 24.8	NA	NA
ERP dispersion (ms)	62.5 ± 37.1	62.5 ± 37.1	NA	NA
Overall LVZ%	21.5 ± 24.2	9.1 ± 12.3	37.6 ± 26.3	<0.001

*AF, atrial fibrillation; ERP, effective refractory period; LA, left atrium; LVEF, left ventricular ejection fraction; LVZ, low-voltage zones*.

### Atrial Fibrillation Inducibility, Sustainability, and Termination

In 71 patients who underwent ERP measurement, AF with any duration could be induced in 30 (42.3%) patients, yet 9 of them were <30 s that was not counted as inducible AF. In the remaining 21 inducible AF, 13 (61.9%) were sustained (Table 2). The coupling interval that induced AF was 179 ± 28 ms. The percentages of AF induced in the right PV ostium, left PV ostium, and CS ostium were 56.7, 40.3, and 3.0%, respectively. Neither atrial flutter nor paroxysmal supraventricular tachycardia was induced.

**Table 2:** Comparison between patients with and without inducible, sustained, and terminable atrial fibrillation (AF).

	**Inducible AF**			**Sustained AF**			**Terminable AF**		
	**With (*n* = 21)**	**Without(*n* = 50)**	***p***	**With(*n* = 13)**	**Without (*n* = 8)**	***p***	**With (*n* = 21)**	**Without(n = 34)**	***p***
Age (years)	65.2 ± 10.6	68.3 ± 8.6	0.200	69.5 ± 8.1	58.3 ± 11.2	0.016	63.3 ± 9.3	65.8 ± 12.9	0.445
Female (%)	8 (38.1%)	26 (52.0%)	0.284	5 (38.5%)	3 (37.5%)	1.000	6 (28.6%)	10 (29.4%)	0.947
Persistent AF (%)	1 (4.8%)	5 (10.0%)	0.665	1 (7.7%)	0	1.000	16 (76.1%)	26 (76.5%)	0.981
CHA_2_DS_2_-VASc	3.6 ± 1.7	3.6 ± 1.8	0.964	4.3 ± 1.7	2.6 ± 1.1	0.037	2.7 ± 1.5	3.1 ± 1.7	0.306
LA volume (ml)	157.9 ± 36.0	132.6 ± 41.1	0.017	166.4 ± 39.6	143.9 ± 25.6	0.131	151.9 ± 34.1	155.8 ± 29.4	0.655
LA diameter (mm)	40.4 ± 3.9	39.3 ± 5.4	0.417	40.6 ± 4.4	39.8 ± 2.9	0.651	44.9 ± 5.1	44.2 ± 4.9	0.611
ERP mean (ms)	198 ± 25	220 ± 21	<0.001	205 ± 18	187 ± 32	0.172	NA	NA	NA
ERP dispersion (ms)	89 ± 30	51 ± 34	<0.001	80 ± 27	104 ± 30	0.071	NA	NA	NA
Overall LVZ %	10.2 ± 11.8	8.5 ± 12.6	0.606	16.2 ± 11.5	0.5 ± 0.7	<0.001	26.3 ± 22.3	44.6 ± 26.4	0.011

*Abbreviation the same as Table 1*.

In 55 patients who underwent ibutilide termination protocol, ibutilide was successfully administered in all patients without Torsade de points. AF was terminated in 21 (38.2%) patients (Table 2). The duration between cardioversion and initiation ibutilide administration was 17.6 ± 9.5 min.

### Low-Voltage Zone Size, Distribution, and Risk Factors

An average of 932 points were taken per map. The average LA surface area and volume were 86.06 cm^2^ and 146.2 ml, respectively. LVZ presence was found in 103 (81.7%) patients. Patients with persistent AF, larger LA volume, and longer ERP mean tended to present LVZ. After adjusting for persistent AF and LA volume, only longer ERP dispersion remained the significant risk factor for LVZ presence (Table 3).

**Table 3:** Risk factors for the presence of low-voltage zones (LVZ).

	**Univariate analysis**			**Multivariate analysis**		
	**OR**	**95% CI**	***p***	**OR**	**95% CI**	***p***
Age (years)	1.032	0.990–1.076	0.137			
Female	1.638	0.621–4.324	0.319			
Persistent AF	5.380	1.505–19.238	0.010	1.742	0.175–17.31	0.636
CHA_2_DS_2_-VASc	1.151	0.884–1.498	0.297			
LA diameter (mm)	1.067	0.981–1.161	0.130			
LA volume (ml)	1.016	1.001–1.031	0.035	1.018	1.000–1.036	0.052

*Abbreviation the same as Table 1*.

Overall LVZ% was 21.5 ± 24.2% (Table 1). Patients who underwent ibutilide termination protocol had higher overall LVZ% and larger LVZ% in all the LA segments compared with patients who underwent induction protocol (Table 1 and Figure 2C). The LVZ distribution was heterogeneous. In general, segmental LVZ% was highest in the septum but lowest in the lateral wall (Figure 2C).

### Association of Overall Low-Voltage Zone Quantity With Atrial Fibrillation Dynamics

The overall LVZ% had no significant difference between patients with and without inducible AF (Table 2). After adjusting for ERP mean and LA volume, only ERP dispersion remained as the risk factor for inducible AF (OR 1.021, 95% CI 1.004–1.039, *p* = 0.018). However, in patients with sustainable AF over 300 s, overall LVZ% was significantly larger (Table 2). Multivariate analysis was not performed for risk factors of sustainable AF, due to a limited number of sustainable AF patients. Higher degree of overall LVZ% was the only risk factor for interminable AF (OR 0.015, 95% CI 1.006–1.055, *p* = 0.015).

### Association of Low-Voltage Zone Distribution With Atrial Fibrillation Dynamics

**Figure 3:**
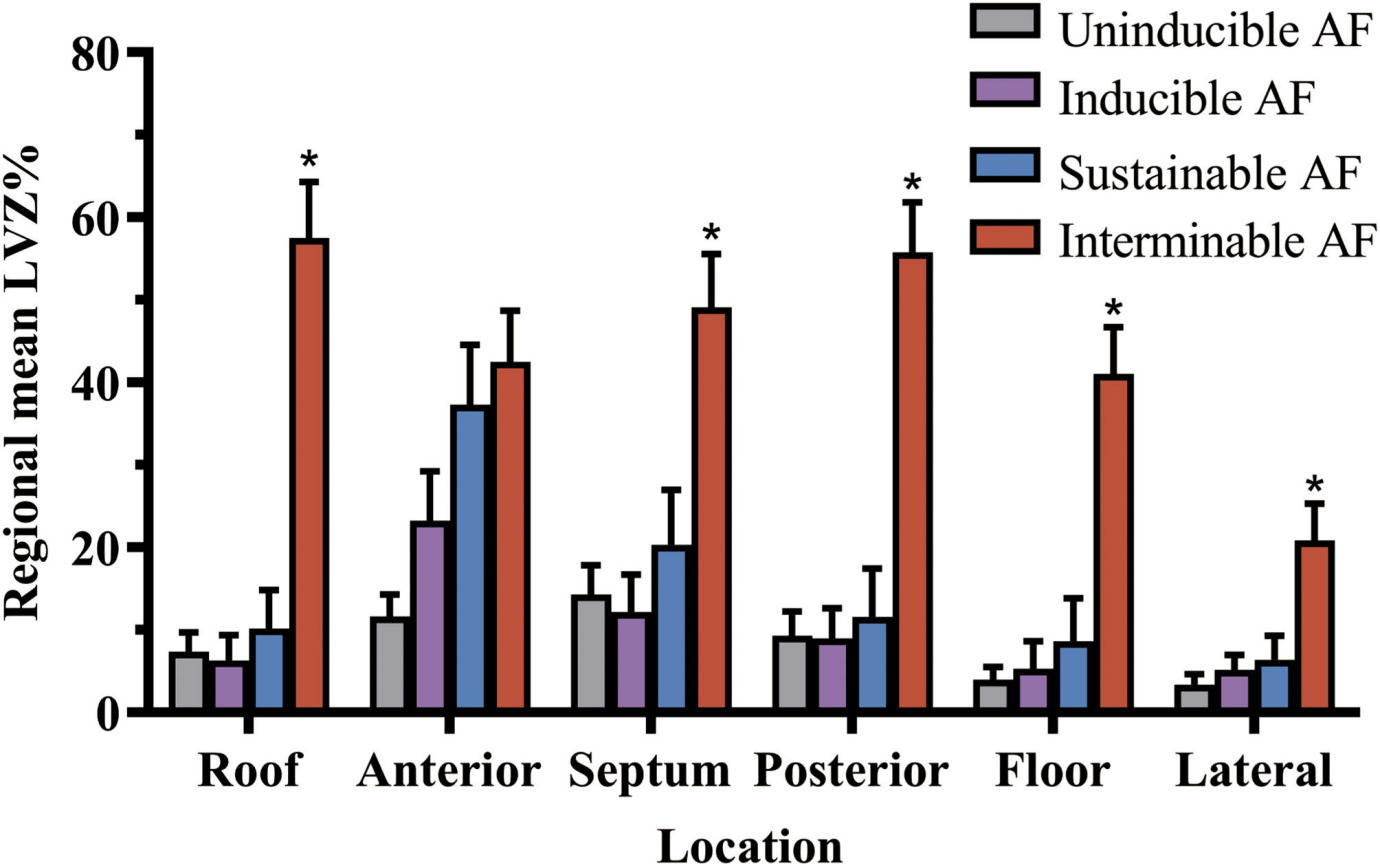
The LVZ distribution of left atrium in the different stages of AF. LVZ% gradually enlarged as AF turned from uninducible into sustainable and interminable. *Compared between sustained and interminable AF patients, *p* < 0.05. The bar charts were drawn using means and standard error. AF, atrial fibrillation; LVZ, low voltage zones.

Compared with uninducible AF patients, inducible AF patients had higher LVZ% in the anterior wall (Figure 2D). Sustainable AF patients had larger LVZ% in areas including the anterior wall and the septum, compared with those with unsustained AF (Figure 2E). By contrast, interminable AF patients exhibited larger LVZ% in the roof, anterior wall, posterior wall, and floor than terminable AF patients (Figure 2F). In addition, except for the anterior wall, interminable AF patients had a higher degree of LVZ% in all segments than sustainable AF patients (Figure 3).

## Discussion

Our study had the following major findings: (1) ERP dispersion rather than overall LVZ% was correlated with inducible AF. (2) Larger overall LVZ% promoted AF to be sustainable and interminable. (3) The segmental LVZ distribution pattern was diverse in different stages of AF. Segmental LVZ% difference was initially observed in the anterior wall for patients with inducible AF, and the septum was further affected in patients with sustainable AF, and the roof, posterior wall, and floor were finally affected in those with interminable AF. The lateral wall was the least affected segment in all stages of AF.

It is reported that the causes of LVZ in AF were correlated with scars, epicardial adipose tissue, and contact areas of neighboring structures (14–16). The association between AF inducibility and LVZ has been in dispute. Masuda et al. (17) demonstrated that AF was usually induced in the atrium with higher LVZ%. In contrast, Kosiuk et al. (18) found that new appearing LVZ was not associated with inducibility of AF in patients with redo-ablation, which was parallel to our findings that overall LVZ% was similar between patients with and without inducible AF. The different duration used for the definition of inducible AF might account for such dispute (17, 18). However, we found that overall LVZ% was larger for patients with sustained AF using the cutoff value of 300 s, indicating that the LVZ% was associated with AF perpetuation rather than inducibility. Similar findings were reported in a study conducted on hypertensive patients without AF, that patients with higher overall LVZ% were inclined to induce sustainable AF (19). Generally, LVZ was associated with fragmented signal and slow conduction (20). This could help to facilitate reentry sustainability by increasing the excitable gap between wave-fronts and wave-tails.

Ibutilide prolongs the phase 3 of cardiac action potential, resulting in increased refractoriness of atrial myocytes and promoting reentry conduction block. The electric activation during AF would be unstable after ibutilide administration (21). Gradually, AF could be terminated owing to the intensification of spiral wave drifting and collision (22). Low-dose ibutilide can decrease negative complex fractionated atrial electrogram (CFAE), which is not related to the occurrence of AF, while displaying positive CFAE. Our previous study found that interminable AF during ibutilide-guided CFAE ablation predicted poor outcome after ablation, suggesting more complex substrates in ibutilide interminable AF patients (23). In our present study, larger overall LVZ% was found in interminable AF patients. In a scarred atrium, LVZ could act as the anchoring site for spiral waves, decreasing spiral drifting and preventing AF termination (24, 25).

The LVZ distribution was diverse during different stages of AF. We found that inducible AF patients had higher segmental LVZ% presented in the anterior wall. With the stage of AF advanced, the LVZ presented in the LA was gradually extended to the septum, roof, and posterior wall. A study observed that a consistent, complete, or near-complete line of block extended from the oval fossa and septal mitral annular region, passing posteriorly around the right inferior PV, and then reaching the posterior wall (75% of cases rising to the LA roof) in patients with spontaneous AF (26). This septopulmonary line of block appeared critical to the formation and interaction of wavefronts in the evolution from initiation to establishment of AF in all cases, while other functional line of blocks (especially on the anterior wall), not seen during sinus rhythm, were formed during initiating sequences in certain cases (26). Therefore, higher LVZ% in the anterior wall could facilitate the line of block, which further promotes the inducible AF. However, we found no significant difference in segmental LVZ% presented in the anterior wall between interminable and sustained AF patients, while interminable AF patients exhibited a higher segmental LVZ% presented in the posterior wall. A previous study found that PVI plus the isolation of the posterior wall facilitated AF termination, suggesting great contribution of the posterior wall in AF termination (27). Phase mapping of the LA rotor and wavelets in persistent AF indicated that the isolation of the posterior wall not only reduced the critical mass for maintenance of AF within the posterior wall but also decreased the rotors and multiple wavelets in the other regions (28). Thus, the LVZ of the posterior wall might play a more important role compared with the anterior wall in the advanced stage of AF.

### Clinical Implications

The present study gives a deeper look at the relationship between LVZ and different stages of AF. In the present study, overall LVZ% gradually enlarged as AF turned from uninducible into sustainable and interminable. Initial segmental LVZ% difference was observed in the anterior wall for inducible AF, which then affected the septum in sustained AF, and further affected the posterior wall and floor in interminable AF. As for ablation strategy, the anterior line of AF crossing the anterior wall scar would be reasonable for the early stage of AF modification, while the posterior wall isolation would be advised for the advanced stage of AF. On the contrary, the lateral wall was the least affected segment with LVZ. Thus, ablation in the lateral wall needs cautious considerations.

### Limitations

There are several limitations of our study. First, the impact of anatomic change in the atrium on LVZ cannot be determined as we lack imaging and biological proof. Second, the bipolar voltage of the right atrium was not collected, which prohibited further analysis of the contribution of right atrial LVZ in AF initiation, sustainability, and termination. Further studies are needed to elucidate such association. Third, patients with prior ablation, previous radiotherapy or chemotherapy, and surgeries in the heart, lungs, and esophagus were not investigated in this study. Substrates underlying AF initiation, perpetuation, and termination may be different. In addition, different pacing protocols, varying pacing outputs, and different pacing sites could result in different relationships between LVZ with AF initiation, perpetuation, and termination. A previous report found that rapid pacing protocol could result in AF inducibility in 25–67% of people without history of AF, which could be unspecific to assess AF inducibility (29, 30). According to previous works, we assessed the AF inducibility with the extra stimulus protocol (9, 11, 31). Further studies are needed to elucidate different pacing protocols and sites on these relationships. Finally, although comparable with previous studies, the sample size of our study is relatively small (9, 11, 31). Studies that include a larger population might be needed to validate our findings.

## Conclusion

The association of LVZ with AF initiation, sustaining, and termination was different depending on its size and distribution. These findings would provide evidence when modifying the strategies for AF ablation. Special attention should be paid to the size and distribution of LVZ such as considering its associations with AF initiation, perpetuation, and termination.

## Data Availability Statement

The original contributions presented in the study are included in the article/supplementary material, further inquiries can be directed to the corresponding author.

## Ethics Statement

The studies involving human participants were reviewed and approved by Ethics Committee of the Beijing Chaoyang Hospital. The patients/participants provided their written informed consent to participate in this study.

## Author Contributions

ZL, YX, XYi, and XYa were responsible for the conception and design of the study. ZL, YX, CG, XL, PF, and XYi contributed substantially to the data acquisition. ZL, YX, XL, PF, XYi, and XYa were part of the data analysis committee. ZL, YX, CG, XL, PF, XYi, and XYa contributed to the data interpretation. CG and XL were responsible for acquisition of the survival data and comorbidities. ZL and YX drafted the manuscript. All authors contributed substantially to the critical revising of the manuscript for important intellectual content, to the final approval of the version to be published, and agreed to be accountable for all aspects of the work.

## Conflict of Interest

The authors declare that the research was conducted in the absence of any commercial or financial relationships that could be construed as a potential conflict of interest.

## Publisher's Note

All claims expressed in this article are solely those of the authors and do not necessarily represent those of their affiliated organizations, or those of the publisher, the editors and the reviewers. Any product that may be evaluated in this article, or claim that may be made by its manufacturer, is not guaranteed or endorsed by the publisher.

## References

[1] Kirchhof P Benussi S Kotecha D Ahlsson A Atar D Casadei B. 2016 ESC Guidelines for the management of atrial fibrillation developed in collaboration with EACTS. Eur Heart J. (2016) 37:2893–962. 10.5603/KP.2016.0172 27567408

[2] Wang XH Li Z Mao JL Zang MH Pu J. Low voltage areas in paroxysmal atrial fibrillation: the prevalence, risk factors and impact on the effectiveness of catheter ablation. Int J Cardiol. (2018) 269:139–44. 10.1016/j.ijcard.2018.07.076 30060968

[3] Marrouche NF Wilber D Hindricks G Jais P Akoum N Marchlinski F. Association of atrial tissue fibrosis identified by delayed enhancement MRI and atrial fibrillation catheter ablation: the DECAAF study. JAMA. (2014) 311:498–506. 10.1001/jama.2014.3 24496537

[4] Boldt A Wetzel U Lauschke J Weigl J Gummert J Hindricks G. Fibrosis in left atrial tissue of patients with atrial fibrillation with and without underlying mitral valve disease. Heart. (2004) 90:400–5. 10.1136/hrt.2003.015347 15020515 PMC1768173

[5] Chen J Arentz T Cochet H Muller-Edenborn B Kim S Moreno-Weidmann Z. Extent and spatial distribution of left atrial arrhythmogenic sites, late gadolinium enhancement at magnetic resonance imaging, and low-voltage areas in patients with persistent atrial fibrillation: comparison of imaging vs. electrical parameters of fibrosis and arrhythmogenesis. Europace. (2019) 21:1484–93. 10.1093/europace/euz159 31280323

[6] Jadidi AS Lehrmann H Keyl C Sorrel J Markstein V Minners J. Ablation of persistent atrial fibrillation targeting low-voltage areas with selective activation characteristics. Circ Arrhythm Electrophysiol. (2016) 9:e002962. 10.1161/CIRCEP.115.002962 26966286

[7] Blandino A Bianchi F Grossi S Biondi-Zoccai G Conte MR Gaido L. Left atrial substrate modification targeting low-voltage areas for catheter ablation of atrial fibrillation: a systematic review and meta-analysis. Pacing Clin Electrophysiol. (2017) 40:199–212. 10.1111/pace.13015 28054377

[8] Dinov B Kosiuk J Kircher S Bollmann A Acou WJ Arya A. Impact of metabolic syndrome on left atrial electroanatomical remodeling and outcomes after radiofrequency ablation of nonvalvular atrial fibrillation. Circ Arrhythm Electrophysiol. (2014) 7:483–9. 10.1161/CIRCEP.113.001185 24833645

[9] Sanders P Morton JB Davidson NC Spence SJ Vohra JK Sparks PB. Electrical remodeling of the atria in congestive heart failure: electrophysiological and electroanatomic mapping in humans. Circulation. (2003) 108:1461–8. 10.1161/01.CIR.0000090688.49283.67 12952837

[10] Oliveira M da Silva MN Timoteo AT Feliciano J Sousa L Santos S. Inducibility of atrial fibrillation during electrophysiologic evaluation is associated with increased dispersion of atrial refractoriness. Int J Cardiol. (2009) 136:130–5. 10.1016/j.ijcard.2008.04.097 18676037

[11] Yin X Zhao Y Xi Y Cheng N Xia Y Zhang S. The early stage of the atrial electroanatomic remodeling as substrates for atrial fibrillation in hypertensive patients. J Am Heart Assoc. (2014) 3:e001033. 10.1161/JAHA.114.001033 25237045 PMC4323835

[12] Jais P Hocini M Hsu LF Sanders P Scavee C Weerasooriya R. Technique and results of linear ablation at the mitral isthmus. Circulation. (2004) 110:2996–3002. 10.1161/01.CIR.0000146917.75041.58 15520313

[13] Vlachos K Efremidis M Letsas KP Bazoukis G Martin R Kalafateli M. Low-voltage areas detected by high-density electroanatomical mapping predict recurrence after ablation for paroxysmal atrial fibrillation. J Cardiovasc Electrophysiol. (2017) 28:1393–402. 10.1111/jce.13321 28884923

[14] Qureshi NA Kim SJ Cantwell CD Afonso VX Bai W Ali RL. Voltage during atrial fibrillation is superior to voltage during sinus rhythm in localizing areas of delayed enhancement on magnetic resonance imaging: an assessment of the posterior left atrium in patients with persistent atrial fibrillation. Heart Rhythm. (2019) 16:1357–67. 10.1016/j.hrthm.2019.05.032 31170484 PMC6722483

[15] Klein C Brunereau J Lacroix D Ninni S Brigadeau F Klug D. Left atrial epicardial adipose tissue radiodensity is associated with electrophysiological properties of atrial myocardium in patients with atrial fibrillation. Eur Radiol. (2019) 29:3027–35. 10.1007/s00330-018-5793-4 30402702

[16] Nakahara S Hori Y Nishiyama N Okumura Y Fukuda R Kobayashi S. Influence of the left atrial contact areas on fixed low-voltage zones during atrial fibrillation and sinus rhythm in persistent atrial fibrillation. J Cardiovasc Electrophysiol. (2017) 28:1259–68. 10.1111/jce.13301 28727202

[17] Masuda M Fujita M Iida O Okamoto S Ishihara T Nanto K. Influence of underlying substrate on atrial tachyarrhythmias after pulmonary vein isolation. Heart Rhythm. (2016) 13:870–8. 10.1016/j.hrthm.2015.12.029 26711800

[18] Kosiuk J Grundig S Dinov B Mussigbrodt A Richter S Sommer P. Significance of inducibility of atrial fibrillation after pulmonary vein isolation in patients with healthy left atrium substrate. J Cardiovasc Electrophysiol. (2019) 30:2767–72. 10.1111/jce.14234 31626352

[19] Medi C Kalman JM Spence SJ Teh AW Lee G Bader I. Atrial electrical and structural changes associated with longstanding hypertension in humans: implications for the substrate for atrial fibrillation. J Cardiovasc Electrophysiol. (2011) 22:1317–24. 10.1111/j.1540-8167.2011.02125.x 21736657

[20] Roberts-Thomson KC Kistler PM Sanders P Morton JB Haqqani HM Stevenson I. Fractionated atrial electrograms during sinus rhythm: relationship to age, voltage, and conduction velocity. Heart Rhythm. (2009) 6:587–91. 10.1016/j.hrthm.2009.02.023 19329365

[21] Biviano AB Ciaccio EJ Gabelman T Whang W Garan H. Ibutilide increases the variability and complexity of atrial fibrillation electrograms: antiarrhythmic insights using signal analyses. Pacing Clin Electrophysiol. (2013) 36:1228–35. 10.1111/pace.12224 23875908 PMC3809098

[22] Nakagawa H Honjo H Ishiguro YS Yamazaki M Okuno Y Harada M. Acute amiodarone promotes drift and early termination of spiral wave re-entry. Heart Vessels. (2010) 25:338–47. 10.1007/s00380-009-1184-8 20676844

[23] Sun XR Tian Y Shah A Yin XD Shi L Wang YJ. Low-dose ibutilide combined with catheter ablation of persistent atrial fibrillation: procedural impact and clinical outcome. Cardiol Res Pract. (2019) 2019:3210803. 10.1155/2019/3210803 30719341 PMC6334335

[24] Angel N Kholmovski EG Ghafoori E Dosdall DJ MacLeod RS Ranjan R. Regions of high dominant frequency in chronic atrial fibrillation anchored to areas of atrial fibrosis. Comput Cardiol. (2019) 46:10.22489/cinc.2019.403. 10.22489/cinc.2019.403 32161769 PMC7065674

[25] Haissaguerre M Shah AJ Cochet H Hocini M Dubois R Efimov I. Intermittent drivers anchoring to structural heterogeneities as a major pathophysiological mechanism of human persistent atrial fibrillation. J Physiol. (2016) 594:2387–98. 10.1113/JP270617 26890861 PMC4850206

[26] Jones DG Markides V Chow AWC Schilling RJ Kanagaratnam P Wong T. Characterization and consistency of interactions of triggers and substrate at the onset of paroxysmal atrial fibrillation. Europace. (2017) 19:1454–62. 10.1093/europace/euw229 28339601

[27] Kumagai K Toyama H Zhang B. Effects of additional ablation of low-voltage areas after Box isolation for persistent atrial fibrillation. J Arrhythm. (2019) 35:197–204. 10.1002/joa3.12169 31007783 PMC6457373

[28] Kumagai K Toyama H Ashihara T. Impact of box isolation on rotors and multiple wavelets in persistent atrial fibrillation. Circ J. (2020) 84:419–26. 10.1253/circj.CJ-19-0826 32051349

[29] Huang W Liu T Shehata M Zhang K Yao Y Niu G. Inducibility of atrial fibrillation in the absence of atrial fibrillation: what does it mean to be normal? Heart Rhythm. (2011) 8:489–92. 10.1016/j.hrthm.2010.11.036 21111062

[30] Razavi M Cheng J Rasekh A Yang D Delapasse S Ai T. Slow pathway ablation decreases vulnerability to pacing-induced atrial fibrillation: possible role of vagal denervation. Pacing Clin Electrophysiol. (2006) 29:1234–9. 10.1111/j.1540-8159.2006.00528.x 17100676

[31] Williams SE Linton NWF Harrison J Chubb H Whitaker J Gill J. Intra-atrial conduction delay revealed by multisite incremental atrial pacing is an independent marker of remodeling in human atrial fibrillation. JACC Clin Electrophysiol. (2017) 3:1006–17. 10.1016/j.jacep.2017.02.012 28966986 PMC5612260

